# Developing lightweight structural concrete with enhanced thermal and durability properties through nano-silica and expanded polystyrene integration

**DOI:** 10.1038/s41598-025-11354-7

**Published:** 2025-07-25

**Authors:** Sabry A. Ahmed, Esraa Ebrahem, Ahmed A. M. El-Amir, M. S. El-Feky

**Affiliations:** 1 Faculty of Engineering, Zagazig University, Zagazig, Egypt; 2 Zagazig University, Zagazig, Sharkia Egypt; 3 Refractory and Ceramic Materials Department, Central Metallurgical Research and Development Institute, Cairo, Egypt; 4 Department of Civil Engineering, National Research Centre, Cairo, Egypt

**Keywords:** Lightweight structural concrete, Nano-silica, Expanded polystyrene (EPS), Thermal conductivity, Water permeability, Sustainable building materials, Engineering, Materials science, Nanoscience and technology

## Abstract

This comprehensive study investigates the development of lightweight structural concrete with enhanced thermal and durability properties by strategically incorporating nano-silica (NS) and expanded polystyrene (EPS) granules. This research aims to design a high-performance concrete composite that can achieve superior thermal insulation, improved water permeability, and maintain structural integrity. NS was strategically incorporated at varying dosages of 0.75, 1, and 1.25% by weight of cement, while EPS was used to replace fine aggregates at 25, 50, 75, and 100% replacement levels. The thermal performance of the concrete mixtures was systematically evaluated using the advanced transient plane source method, providing insights into thermal conductivity, thermal diffusivity, and volumetric heat capacity. The experimental results demonstrate that the addition of NS led to a significant 15% reduction in thermal conductivity, attributed to the filler effect and pozzolanic reactivity of nano-silica. The incorporation of EPS granules exhibited an even more pronounced impact, decreasing the thermal conductivity of concrete by up to 80.5% as the replacement level increased. Notably, the combined use of NS and EPS resulted in a synergistic effect, achieving a remarkable 39–86% reduction in thermal conductivity, 28–71%, and 28–79% reductions in thermal effusivity and diffusivity, respectively, compared to the control mix. Furthermore, the optimal NS content of 1–1.25% was found to enhance the compressive strength by up to 36.5% and reduce the water permeability by 40–52%, indicating improved mechanical and durability properties. These findings highlight the transformative potential of this composite material in developing high-performance, thermally-efficient, and sustainable concrete for energy-efficient buildings, reducing operational energy demands and carbon footprints.

## Introduction

The advancement of high-performance concrete composites with superior thermal insulation and structural integrity has emerged as a vital research focus within the construction industry. This is largely driven by the increasing demand for sustainable, energy-efficient building materials capable of addressing the challenges posed by climate change, rising energy costs, and the goal of achieving net-zero emissions.

Minimizing energy consumption in buildings has become a paramount objective for engineers and researchers, motivated by the need to reduce environmental pollution and lower the economic burden associated with fuel consumption^1^. Currently, the building sector is responsible for approximately 30% of global energy consumption and contributes around 27% of CO_2_ emissions^2^. These figures innovative building materials that possess enhanced thermal insulation properties and improved mechanical characteristics, thereby facilitating reductions in energy consumption, operational costs, and the environmental impact of the construction industry^3^.

Although concrete is widely used and exhibits excellent structural properties, it has relatively high thermal conductivity, which can result in considerable heat transfer through building envelopes. This characteristic leads to increased energy demands for heating and cooling, subsequently raising operational costs and raising environmental concerns. Consequently, the development of thermally efficient concrete has become a priority in materials research.

The integration of insulating materials into concrete mixtures has garnered significant attention as a viable strategy to enhance thermal insulation while preserving adequate mechanical properties. Among these materials, nanomaterial and expanded polystyrene (EPS) have demonstrated promising results in several studies. Nanoparticles, with their high surface area-to-volume ratio and unique Nano scale properties, can markedly influence the microstructure and thermal behavior of cementitious materials^4^. In contrast, EPS, characterized by its closed-cell structure and low density, provides excellent insulation properties when utilized as a lightweight aggregate in concrete^5,6^.

Researchers have investigated the application of nanotechnology, particularly through the addition of nano-silica (SiO_2_), to enhance the performance of lightweight concrete that incorporates EPS^7–9^.

Recent studies have explored various methodologies to improve the thermal performance of concrete. Wang et al.^10^ assessed the effects of nanoparticles on concrete’s compressive strength and thermal conductivity, finding that a 0.3% substitution of cement with nano-clay resulted in an increase in the thermal conductivity coefficient. Saleh et al.^11^ examined the combined impact of nano-silica and polystyrene granules on concrete properties, achieving thermal conductivity values ranging from 0.43 to 0.45 W/m °C.

Lightweight aggregate (LWA) concrete has been shown to possess lower thermal conductivity compared to normal-weight concrete due to its increased porosity^12–14^. This characteristic contributes to a lower total heat transfer coefficient in LWA concrete envelopes, thereby enhancing overall thermal resistance. Dixit et al.^15^ developed a lightweight EPS-cement composite with a compressive strength of 45 MPa, a density of 1677 kg/m^3^, and a thermal conductivity of 0.58 W/m K, demonstrating the potential for balancing structural requirements with thermal efficiency.

Alternative strategies have also been examined, such as the incorporation of recycled materials into concrete mixtures. Priya et al.^16^ partially replaced fine aggregates with wood waste (sawdust) at ratios of 5%, 10%, and 15% by weight. Boussetoua et al.^17^ found that reducing the percentage of polystyrene in concrete mixtures resulted in increased thermal conductivity while enhancing mechanical properties.

The utilization of EPS waste in lightweight mortar and masonry units has been explored by Ali et al.^18^. Their study incorporated EPS at dosages of 0, 10, 15, 20, and 26 kg/m^3^ as a partial sand replacement, alongside silica fume to improve mechanical properties. The results indicated a decrease in density, compressive strength, tensile strength, static modulus of elasticity, and thermal conductivity coefficient with increasing EPS content.

Layachi^19^ evaluated the effects of varying volumes of EPS beads (0, 40, 45, 50, 55, 60, 65%) on the thermal and mechanical properties of lightweight earth blocks, reporting improvements in thermal insulation performance of up to 57.36% with 65% EPS bead content compared to control samples. Shi^20^ focused on ultra-lightweight expanded polystyrene foamed concrete (EFC) produced through chemical foaming, demonstrating that an optimal volume of EPS particles could reduce thermal conductivity while minimizing the temperature dependence of this property.

EPS is a lightweight, low-density material characterized by exceptional thermal insulation properties, making it an appealing additive for concrete. However, the inclusion of EPS can often compromise the mechanical properties of concrete, such as compressive strength and durability. A systematic investigation of the synergistic effects arising from the combination of EPS with other additives, such as nano-silica (NS), may offer a solution to this challenge.

Nano-silica is a pozzolanic material that can significantly enhance the mechanical and durability properties of concrete through its filler effect and pozzolanic reactivity. The incorporation of NS has been shown to improve compressive strength, decrease water permeability, and refine the microstructure of the concrete matrix. By combining both EPS and NS in concrete, it is hypothesized that a synergistic effect can be achieved, wherein the enhanced thermal insulation provided by EPS is synergized with the improved mechanical and durability characteristics imparted by NS.

Recent advancements in concrete technology have spotlighted the properties of nano-silica (NS) and plastic-based cement composites, particularly in enhancing mechanical strength and durability while reducing thermal conductivity. Nano-silica, recognized for its pozzolanic activity, significantly improves the compressive strength and microstructure of concrete, leading to reduced permeability and enhanced durability. Simultaneously, the incorporation of plastic materials, such as expanded polystyrene (EPS), into cement composites has gained traction due to its lightweight and excellent thermal insulation properties. However, existing research often focuses on the individual effects of these materials rather than their synergistic interactions. This study distinguishes itself by systematically investigating the combined effects of NS and EPS on lightweight concrete, revealing that their integration not only enhances thermal insulation but also maintains or even improves mechanical properties. By optimizing the proportions of these additives, this research contributes new insights into developing high-performance, eco-friendly building materials. The findings aim to fill existing knowledge gaps regarding the interplay between nano-silica and plastic aggregates, offering valuable information for the design of sustainable construction solutions that meet modern energy efficiency and structural integrity standards.

This systematic investigation into high-performance concrete composites integrating NS and EPS is crucial for the development of innovative building materials that address the urgent need for thermally efficient, structurally sound, and sustainable construction solutions. The findings from this research can contribute to the advancement of green building technologies, reduction of carbon footprints, and enhancement of energy performance in buildings, ultimately facilitating the transition toward a more sustainable built environment. Accordingly, the present study aims to comprehensively evaluate the effects of incorporating expanded polystyrene (EPS) granules and nano-silica (NS) on the thermal properties of lightweight concrete. By systematically varying the proportions of these additives, we seek to optimize the thermal insulation capabilities of concrete while ensuring acceptable mechanical performance. This research supports ongoing efforts to develop sustainable, energy-efficient building materials that can significantly mitigate the environmental impact of the construction industry.

 While this study demonstrates promising results, limitations include the scalability of nano-silica production, long-term durability under cyclic thermal loading, and the economic feasibility of large-scale EPS integration. Future work will address these aspects.

## Material and methods

The scope of this research encompasses experimental investigations and analysis of the thermal properties of lightweight concrete incorporating nano-silica SiO_2_ and recycled EPS in various proportions. The study will focus on assessing the mechanical properties, thermal insulation performance, and durability characteristics of the concrete. The research will explore the impact of different combinations and ratios of nano-silica SiO_2_ and recycled EPS on the compressive strength, density, and thermal conductivity of concrete. The study will contribute to a deeper understanding of the synergistic effects between nano-silica SiO_2_ and recycled EPS in lightweight concrete, providing insights for optimizing their combination and proportion in order to achieve superior performance. The findings of this research will aid in the development of sustainable and high-performance lightweight concrete for a wide range of construction applications.

### Materials

**Fig. 1:**
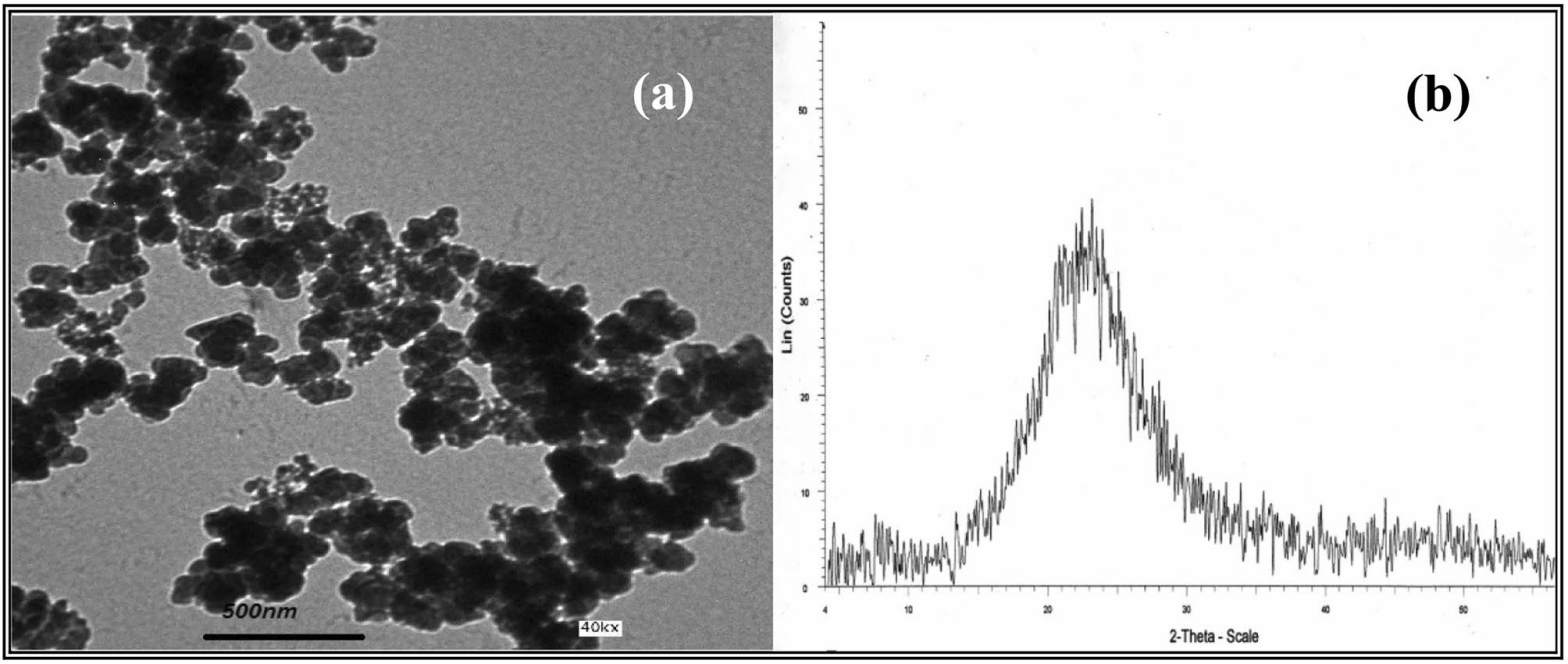
(**a**) TEM micrograph of SiO_2_ Nano particles, (**b**) XRD analysis of SiO_2_ Nano particles.

Ordinary Portland Cement (OPC) conforming to ASTM C150 standard was used as received. Local nano silica was used as a replacement material with 0.75, 1 and 1.25% from cement weight; the properties of SiO_2_ nano particles are shown in Table 1. Transmission electron micrographs (TEM) and powder X-ray diffraction (XRD) diagrams of SiO_2_ nano particles are shown in Fig. 1. The expanded polystyrene foam (EPS) was used as a replacement material with 25, 50, 75 and 100 with sand volume; its characteristics are shown in Table 2. It has a very low density (20–30 kg/m^3^).The sand used in mortars is the locally available sand free from alkali-reactive materials. The water used is the potable water from the water-supply network system, free from suspended solid and organic materials, which can affect the properties of the fresh and hardened concrete.

**Table 1:** Chemical composition and purity of Nano-SiO₂ (wt%).

Element	SiO_2_	Fe_2_O_3_	Al_2_O_3_	MgO	CaO	Na_2_O	P_2_O_5_
NS	99.15	0.06	0.13	0.11	0.14	0.40	0.01

**Table 2:** Chemical and physical properties of EPS foam.

Molecular weight	Density (kg/m^3^)	Beads diameter (mm)	Thermal conductivity W/(m K)	Compressive strength (N/mm^2^)
300.000	17	1.5 3	0.04	0.1

### Mixture proportions (experimental method)

As shown in Table 3, a total of 20 mixtures were prepared using a consistent cement to sand ratio of 1:2. A constant water/cement ratio of 0.50 was maintained throughout the experiment.

**Table 3:** Mixing design of EPS foamed concrete with nano silica SiO2.

Mix name	Cement (kg)	Sand (cm^3^) |	EPS (cm^3^)	Water (cm^3^)	Nano silica (% by wt. of cement)
Control	1.8	2000	0	900	0
N1	1.8	2000	0	900	0.75
N2	1.8	2000	0	900	1
N3	1.8	2000	0	900	1.25
E25	1.8	1500	500	900	0
E50	1.8	1000	1000	900	0
E75	1.8	500	1500	900	0
E100	1.8	0	2000	900	0
E25N0.75	1.8	1500	500	900	0.75
E25N1	1.8	1500	500	900	1
E25N1.25	1.8	1500	500	900	1.25
E50N0.75	1.8	1000	1000	900	0.75
E50N1	1.8	1000	1000	900	1
E50N1.25	1.8	1000	1000	900	1.25
E75N0.75	1.8	500	1500	900	0.75
E75N1	1.8	500	1500	900	1
E75N1.25	1.8	500	1500	900	1.25
E100N0.75	1.8	0	2000	900	0.75
E100N1	1.8	0	2000	900	1
E100N1.25	1.8	o	2000	900	1.25

The control mix, denoted as Mix C, consisted of sand and cement only, without any EPS replacement or nano-silica addition.

To investigate the effect of nano-silica on the thermal properties of lightweight concrete, three additional mixes, namely N1, N2, and N3, were prepared. These mixes contained the same constituents as Mix C but included nano-silica at 0.75, 1, and 1.25% respectively, as a replacement for the weight of cement.

To study the influence of EPS without nano-silica on the thermal properties of lightweight concrete, mixes E25, E50, E75, and E100 were prepared. These mixes involved replacing 25, 50, 75, and 100% of the sand volume with EPS.

Furthermore, to examine the combined effects of EPS and, additional mixes were prepared. For example, mixes E25N0.75, E25N1, and E25N1.25 contained 25% EPS and 0.75%, 1%, and 1.25% nano-silica, respectively. Similar combinations were prepared for mixes E50N0.75, E50N1, E50N1.25, E75N0.75, E75N1, E75N1.25, E100N0.75, E100N1, and E100N1.25, where the numbers indicate the percentage of EPS and the letters denote the percentage of nano-silica.

The mixing process involved adding the solution to the mixer along with a portion of water and cement, which were mixed for two minutes. The EPS beads were thoroughly mixed with the sand for five minutes. Part of the water was then added to the nano-silica and sprayed during the mixing process to ensure uniform distribution throughout the mixture. Mixing continued until a consistent and homogeneous mixture was achieved. EPS beads were dry-mixed with sand for 5 min. Nano-silica was pre-dispersed in water via ultrasonic treatment (30 min, 40 kHz) to ensure homogeneity.

Test specimens were cast using only hand compaction. The concrete samples were removed from the molds 24 h after casting and placed in a moist curing tank.

### Testing

**Fig. 2:**
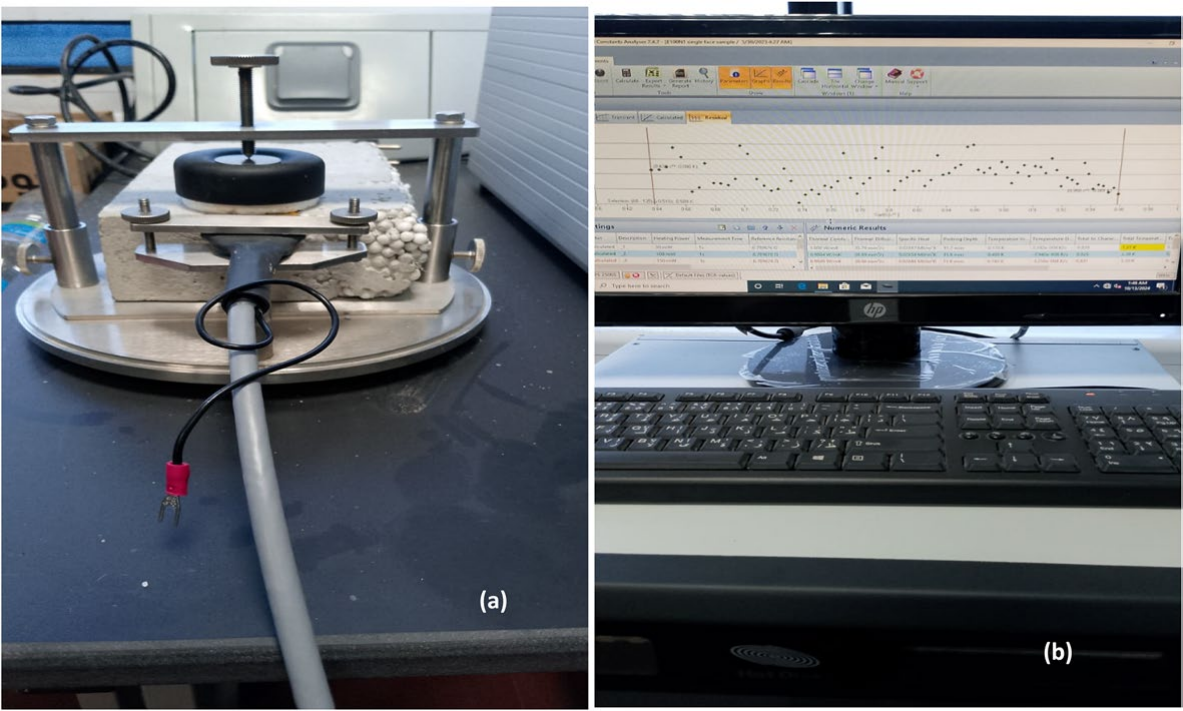
(**a**) and (**b**) Thermal conductivity measurement using the hot disc TPS 2500 S analyzer.

As shown in Fig. 2, thermal properties measurements were performed on the hot disc thermal constants analyzer (Hot Disc TPS 2500 S, Goteborg, Sweden). This Transient plane source (TPS) was the most precise and convenient technique for studying thermal transport properties.

Compressive strength of the cast specimens was determined at a rate of 1.3 mm/min by universal testing machine (Shimadzu, UH-F 1000 KN, Japan).

Mineral composition of nano silica was recognized by an X-ray diffractometer, model: Brukur advanced D8 Kristalloflex (Ni-filtered Cu Kα radiation; λ = 1.544 Å). The microstructure of nano silica was observed by backscattered electron (BSE) in the field emission scanning electron microscopy (FESEM QUANTAFEG 250).

## Results and discussion

### Effect of nano-silica addition on thermal properties

#### Thermal conductivity

**Fig. 3:**
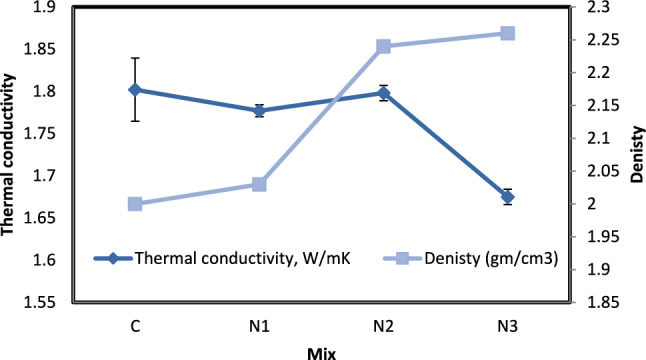
Relation between density and thermal conductivity for mixes with nano silica and without EPS.

As shown in Fig. 3, and Table 4 it has been observed the values of the thermal conductivity coefficient of nano concrete are lower compared to conventional concrete, as the addition of nano-silica material in proportions (0.75, 1, and 1.25%) had a good role in enhancing the ability of thermal insulation. It was also observed that the values of the thermal conductivity coefficient for samples containing nano SiO_2_ ranged between (1.6–1.7) W/m k C, while the values of the thermal conductivity coefficient for conventional concrete samples 1.8 W/m k C. It was also found that the most effective thermal insulation was discovered in (N3) mixture, as the results indicate a decrease in the values of the thermal conductivity coefficient whenever the percentage of adding the nano SiO_2_ increases^11^, this is in line with the outcomes that Jittabuta and Kaya et al. described^21^.

**Table 4:** Effect of nano silica content on the thermal properties of concrete brick.

Sample color	Thermal conductivity, W/mK	Thermal diffusivity, mm^2^/S	Volumetric Specific heat capacity, MJ/m^3^K	Thermal effusivity, Ws¹´²/(m²K)	Denisty (gm/cm^3^)
C	1.802	1.118	1.627	1710.739	2
N1	1.777	1.095	1.631	1701.597	2.03
N2	1.798	1.012	1.779	1788.362	2.24
N3	1.675	1.087	1.547	1609.116	2.26

The reduction in thermal conductivity upon the addition of SiO_2_ can be attributed to several factors. Filler Effect: Nano-silica particles fill the voids between larger aggregates, creating a denser microstructure that reduces pathways for heat conduction. Pozzolanic Reactivity: Nano-silica enhances the pozzolanic reactions within the concrete matrix, leading to the formation of additional calcium silicate hydrates (C–S–H) that improve the material’s overall thermal insulation properties. Air Voids: The incorporation of nano-silica can also lead to increased air void content when being agglomerated especially at high dosages at certain locations within the matrix, which are effective thermal insulators.

Error bars represent ± 1 SD. The Control’s larger variability stems from inherent heterogeneity in non-enhanced concrete.

#### Thermal effusivity

**Fig. 4:**
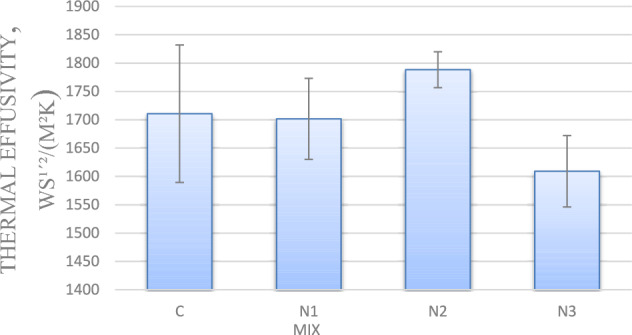
Thermal effusivty for mixes with nano silica and without EPS.

As shown in Fig. 4 for mixes with 0.75, and 1.25% nano-silica, thermal diffusivity values have reduced by 0.5 and 6%, respectively, compared to the control mix. However for mix with 1% nano silica, thermal diffusivity increased by 4%, compared to the control mix.

#### Thermal diffusivity

**Fig. 5:**
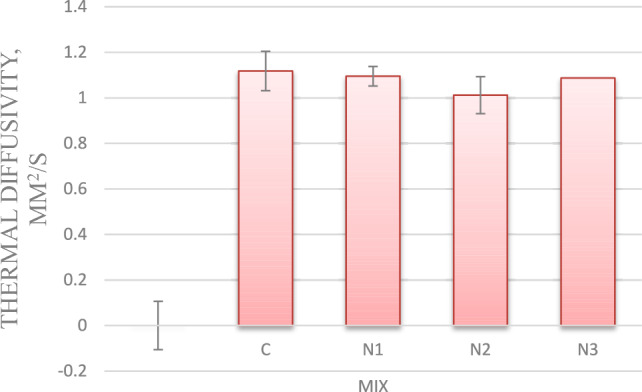
Thermal diffusivity for mixes with nano silica and without EPS.

As shown in Fig. 5 for mixes with 0.75, 1, and 1.25% nano-silica, thermal diffusivity values have reduced by 2, 9, and 14%, respectively, compared to the control mix.

The reduction in thermal diffusivity values is attributed to the reduced TC of concrete. The result is consistent with the study of Saleh et al. (2021).

#### Volumetric heat capacity

**Fig.6:**
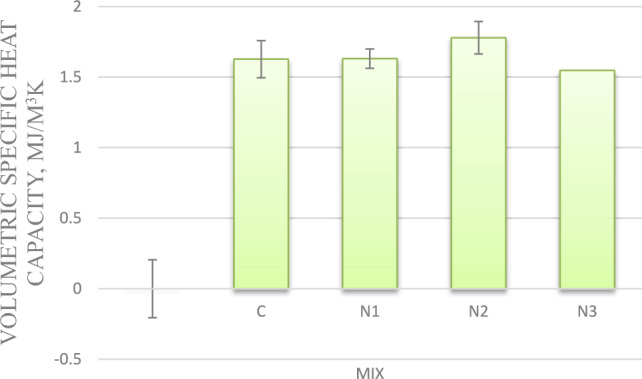
Volumetric heat capacity for mixes with nano silica and without EPS.

For mixes with 0.75%, and 1% nano-silica, Volumetric Specific heat capacity values have increased by 0.5%, and 9%, respectively, compared to the control mix. However for mix with 1.25% nano silica, Volumetric Specific heat capacity decreased by 5%, compared to the control mix as shown in Fig. 6.

### Effect of expanded polystyrene addition on thermal properties

#### Thermal conductivity

**Fig. 7:**
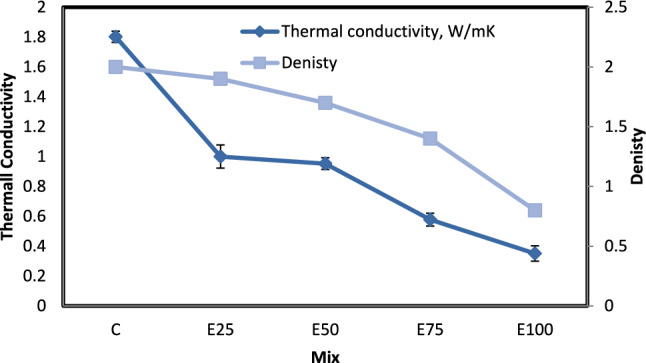
Relation between density and thermal conductivity for mixes with EPS, and without nano silica.

As shown in Table 5, it was noted that the density value decreases as the EPS beads percentage increases. This decrease is attributable to the ultra-lightweight of the EPS beads; the average density of EPS beads particles is 11.4 kg/m^3^. The results of the material’s apparent density are shown in Fig. 7.

**Table 5:** Effect of expanded poly styrene on the thermal properties of concrete brick.

Sample color	Thermal conductivity, W/mK	Thermal diffusivity, mm^2^/S	Volumetric Specific heat capacity, MJ/m^3^K	Thermal effusivity, Ws¹´²/(m²K)	Density (gm/cm^3^)
C	1.802	1.118	1.627	1710.739	2
E25	1.000	0.877	1.214	1093.181	1.9
E50	0.953	0.480	2.024	1385.139	1.7
E75	0.578	0.365	1.609	962.551	1.4
E100	0.351	0.281	1.276	666.222	0.8

From the measurement of thermal conductivity, the thermal insulation quality of the samples was estimated. And the results are explained in Fig. 7. As shown in Table 1 the thermal conductivity coefficient of concrete incorporating EPS are lower compared to conventional concrete, as the addition of EPS material in proportions (25, 50,75, and 100%) had a good role in enhancing the ability of thermal insulation by a ratio (44.5, 47,68 and 80.5%) respectively. The incorporation of 25, 50, 75, and 100% of EPS beads resulted in lower TC by 44.5, 47, 68 and 80 respectively, compared with control samples. This decrease is predicted due to the EPS beads have a lower TC, which ranges between 0.03863 and 0.03365 W/m C◦ as conducted by^22^.

The porosity factor also affects the thermal conductivity of the samples, as closed pores decrease the TC due to the low TC of air^23^. The incorporation of EPS beads elevates porosity by introducing air-filled voids, which concurrently reduces density and thermal conductivity^24^.

#### Thermal effusivity

**Fig. 8:**
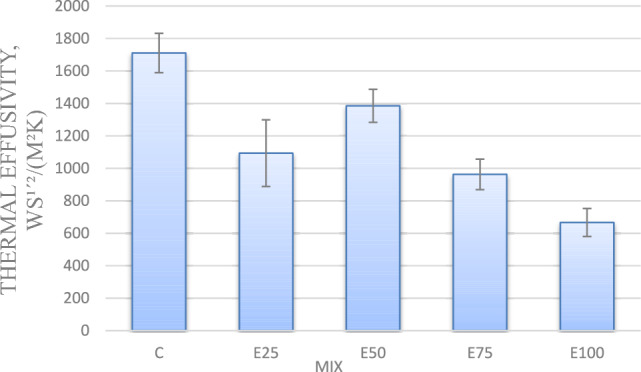
Thermal effusivity for mixes with EPS and without nano silica.

One of the important concepts concerning the efficiency of thermal insulation is thermal effusivity. It characterizes a material’s surface’s capacity to absorb and release heat. Figure 8 demonstrates the variation of thermal effusivity. This Fig. shows that as the EPS beads content increases with 25, 50,75 and 100%, the thermal effusivity of the samples decreases with 39, 19, 45 and 29%, respectively, as compared to the control mix. The same result was declared by Atiki et al.^25^, who investigated compressed earth blocks containing date palm waste aggregates, and those found by Boumhaout et al.^26^, who researched cement mortar incorporating palm mesh fibers.

#### Thermal diffusivity

**Fig. 9:**
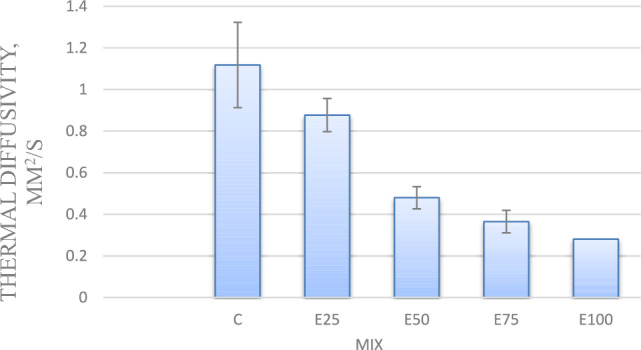
Thermal diffusivity for mixes with EPS and without nano silica.

Figure 9 demonstrates the variation of Thermal diffusivity. This figure shows that as the EPS beads content increases with 25, 50,75 and 100%, the Thermal diffusivity of the samples decreases with 21, 57, 67 and 74%, respectively,.

#### Volumetric heat capacity (VHC)

**Fig. 10:**
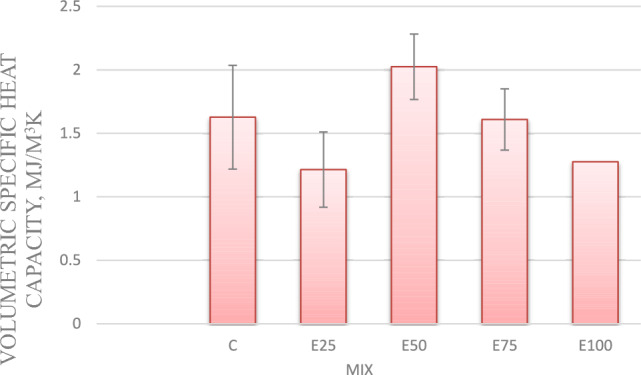
Volumetric specific heat capacity for mixes with EPS and without nano silica.

The VHC is a critical indicator for determining the thermal inertia of building materials. Specific heat capacity evaluation. As shown in Fig. 10 It was noted that when EPS beads rises from 25 to 50%, the specific heat capacity increases from 1.214 to 2.024 MJ/m^3^K by 60%. Then a significant decrease in the specific heat capacity at a content of 75% and 100% of EPS beads. The same behavior was reported by Khoudja et al.^27^. According to Khoudja et al.^27^ this rise in specific heat may be explained by the integration of this particle (EPS beads), whose specific heat is greater than that of the reference sample containing just cement and sand. The reason for the decrease in the specific heat of the samples containing 75 and 100% EPS is due to the enhancement of the porosity (resulting from the increase in the incorporating content of EPS), which is mainly filled with air.

### Effect of combining expanded polystyrene with nano-silica on thermal properties

Tables 6, 7, 8 and 9 Shows the effect of nano silica addition on thermal properties of concrete brick contain different percentages of expanded poly styrene. The combined use of NS and EPS creates a multi-scale insulating structure. NS refines the cement matrix through pozzolanic reactivity and pore-filling, reducing thermal bridging. Simultaneously, EPS introduces macroscopic air voids that disrupt heat transfer pathways. This synergy is quantified by the 39–86% reduction in thermal conductivity, where NS mitigates EPS-induced mechanical weaknesses by enhancing interfacial transition zone (ITZ) bonding. Microstructural analysis (e.g., SEM) confirms denser ITZ around EPS beads in NS-modified mixes, explaining the concurrent strength gain (up to 36.5%) and thermal resistance.

**Table 6:** Effect of nano silica addition on thermal properties of concrete brick contain 25% expanded poly styrene.

Sample color	Thermal conductivity, W/mK	Thermal diffusivity, mm^2^/S	Volumetric Specific heat capacity, MJ/m^3^K	Thermal effusivity, Ws^1^´^2^/(m^2^K)	Denisty (gm/cm^3^)
C	1.802	1.118	1.627	1710.739	2
E25	1.000	0.877	1.214	1093.181	1.9
E25 N0.75	0.970	0.679	1.454	1184.610	1.9
E25 N1	0.962	0.689	1.468	1179.869	2.03
E25 N1.25	1.094	0.804	1.407	1237.254	1.96

**Table 7:** Effect of nano silica addition on thermal properties of concrete brick contain 50% expanded poly styrene.

Sample color	Thermal conductivity, W/mK	Thermal diffusivity, mm^2^/S	Volumetric Specific heat capacity, MJ/m^3^K	Thermal effusivity, Ws^1^´^2^/(m^2^K)	Denisty (gm/cm^3^)
C	1.802	1.118	1.627	1710.739	2
E50	0.953	0.480	2.024	1385.139	1.7
E50 N0.75	0.7512	0.4401	1.7450	1142.1558	1.8
E50 N1	0.7041	0.4112	1.7245	1098.9981	1.7
E50 N1.25	0.6186	0.6223	1.0453	800.0837	1.7

**Table 8:** Effect of nano silica addition on thermal properties of concrete brick contain 75% expanded poly styrene.

Sample color	Thermal conductivity, W/mK	Thermal diffusivity, mm^2^/S	Volumetric Specific heat capacity, MJ/m^3^K	Thermal effusivity, Ws^1^´^2^/(m^2^K)	Denisty (gm/cm^3^)
C	1.802	1.118	1.627	1710.739	2
E75	0.578	0.365	1.609	962.551	1.4
E75 N0.75	0.4674	0.2919	1.6165	867.6986	1.5
E75 N1	0.3949	0.2838	1.4536	753.6764	1.3
E75 N1.25	0.3837	0.4168	0.9795	609.1752	1.5

**Table 9:** Effect of nano silica addition on thermal properties of concrete brick contain 100% expanded poly styrene.

Sample color	Thermal conductivity, W/mK	Thermal diffusivity, mm^2^/S	Volumetric Specific heat capacity, MJ/m^3^K	Thermal effusivity, Ws^1^´^2^/(m^2^K)	Denisty (gm/cm^3^)
C	1.802	1.118	1.627	1710.739	2
E100	0.351	0.281	1.276	666.22	0.8
E100 N0.75	0.2514	0.2743	0.9599	486.83	0.9
E100 N1	0.2887	0.2342	1.2385	597.57	0.98
E100 N1.25	0.2624	0.2575	1.0286	518.72	0.97

#### Thermal conductivity

**Fig. 11:**
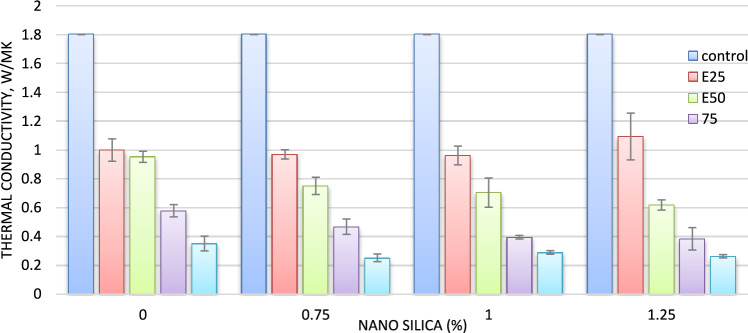
Thermal conductivity of mixes with different replacement of EPS with different percentage of NS.

Table and Fig. 11 shows the combined effect of adding nano-silica and polystyrene granules on the thermal conductivity coefficient of concrete. It was also clear from the results that the combined effect of adding nano silica and polystyrene granules played a role in increasing the concrete’s capacity for thermal insulation, and the thermal conductivity data demonstrate this as compared to conventional concrete. For conventional concrete Adding nano silica in proportions (0.75% and 1%, and 1.25%) to the mixes with 25% EPS decreased in the thermal conductivity coefficient with 46 and 47 and 39% respectively, compared to control mix.

As shown in Fig. 11, adding nano silica with 0.75, 1 and 1.25% to E50 (sample with 50% EPS without nano silica) decreased the thermal conductivity with 58%, 61% and 65%, respectively as compared to |control mix. The same trend was observed with E75 (sample with 75% EPS without nano silica) adding nano silica with 0.75, 1 and 1.25% decreased the thermal conductivity with 74, 78 and 79%, respectively, compared to control mix.

Presence of nano silica with with 0.75, 1, and 1.25% with 100% EPS mix enhanced the thermal insulation with (86, 83 and 85%) compared to E100 mix. illustrates that increasing the amount of polystyrene granules causes a decrease in heat conductivity. The lowest value of the coefficient of thermal conductivity was reached, which varied between (0.262–0.35) W/m k when adding (100%) of EPS to the concrete. The reason for the low thermal conductivity of the concrete is due to the polystyrene granules closing the pores and thus impeding heat transfer^28^. In addition, the decrease in the density of concrete with the increase in the percentage of polystyrene reduces the values of the thermal conductivity coefficient^29^. It was also clear from the results that the combined effect of adding nano silica and polystyrene granules played a role in increasing the concrete’s capacity for thermal insulation, and the thermal conductivity data demonstrate this. Results are compatible with the previous studies. Demirboga^12^ reported that EPA reduced the thermal conductivity of samples up to 43.5%. In this study the maximum reduction due to the EPS was 80% for E100, this is may be due to the lower density of EPS concrete when compared to their study.

#### Thermal effusivity

**Fig. 12:**
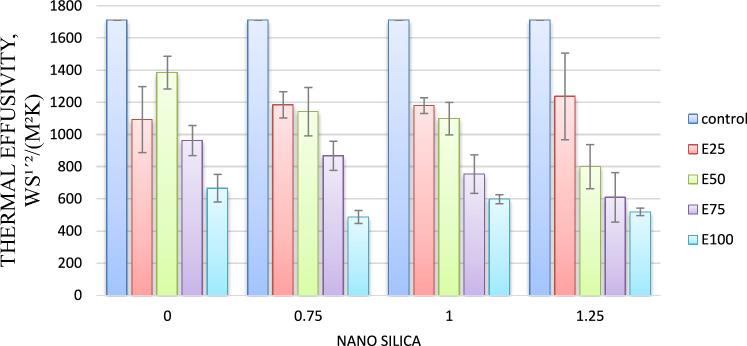
Thermal effusivity of mixes with different replacement of EPS with different percentage of NS.

Figure 12 shows that as Nano silica content increase thermal diffusivity decreased. For mix E25 Adding nano silica with 0.75, 1 and 1.25% resulted in lower thermal effusivity by 30, 31 and 28 respectively, compared to control mix.

As shown in Fig. 12 for mix E50 adding nano silica with 0.75, 1 and 1.25% resulted in lower thermal effusivity by 33, 35 and 53 respectively, compared to control mix.

For mix E75 the same trend was observed adding nano silica with 0.75, 1 and 1.25% resulted in lower thermal effusivity by 49, 56 and 64%, respectively, compared to control mix.

Adding nano silica with 0.75 and 1 and 1.25% to the mix with 100% EPS resulted in decreased the thermal diffusivity by 71, 65 and 69%, respectively compared to control mix.

#### Thermal diffusivity

**Fig. 13:**
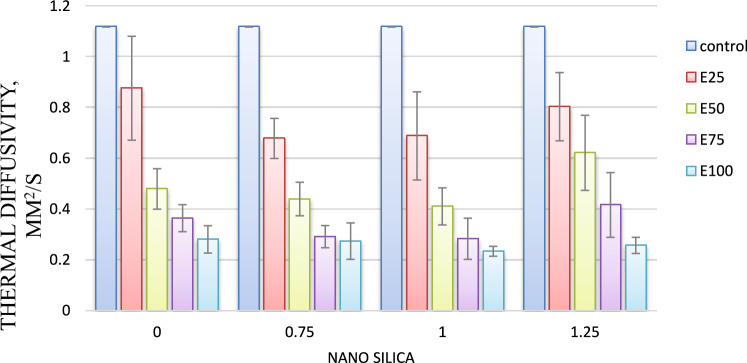
Thermal diffusivity of mixes with different replacement of EPS with different percentage of NS.

For mix with 25% Eps, as Nano silica content increase thermal diffusivity decreased. Adding nano silica with 0.75, 1 and 1.25% resulted in lower thermal diffusivity by 30, 31 and 28 respectively, compared control as shown in Fig. 13.

For mix with 50% Eps, Adding nano silica with 0.75, 1 and 1.25% resulted in lower thermal diffusivity by 33, 35 and 52 respectively, compared control mix as shown in Fig. 13.

For mix with 75% Eps, Adding nano silica with 0.75, 1 and 1.25% resulted in lower thermal diffusivity by 73, 75 and 63 respectively, compared control mix as shown in Fig. 13.

For mix with 100% Eps, Adding nano silica with 0.75, 1 and 1.25% resulted in lower thermal diffusivity by 75, 79 and 76 respectively, compared control mix as shown in Fig. 13.

#### Volumetric specific heat capacity

**Fig. 14:**
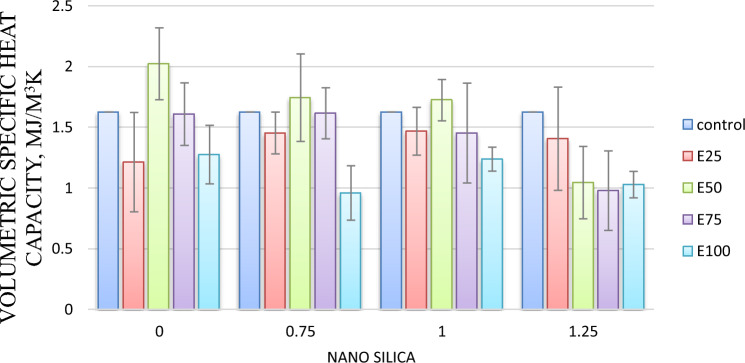
Volumetric specific heat capacity of mixes with different replacement of EPS with different percentage of NS.

For mix with 25% Eps, as Nano silica content increase Volumetric Specific heat capacity decreased. Adding nano silica with 0.75, 1 and 1.25% resulted in lower Volumetric Specific heat capacity by 10, 9 and 13 respectively, compared control mix as shown in Fig. 14

For mix with 50% Eps, Adding nano silica with 0.75, 1 and 1.25% resulted in lower Volumetric Specific heat capacity by 33, 35 and 52 respectively, compared control mix as shown in Fig. 14.

For mix with 75% Eps, Adding nano silica with 0.75, 1 and 1.25% resulted in lower Volumetric Specific heat capacity by 1, 11 and 40%, respectively, compared control mix as shown in Fig. 14.

For mix with 50% Eps, Adding nano silica with 0.75, 1 and 1.25% resulted in lower Volumetric Specific heat capacity by 41, 24 and 37%, respectively, compared control mix as shown in Fig. 14.

#### Compressive strength

**Fig. 15:**
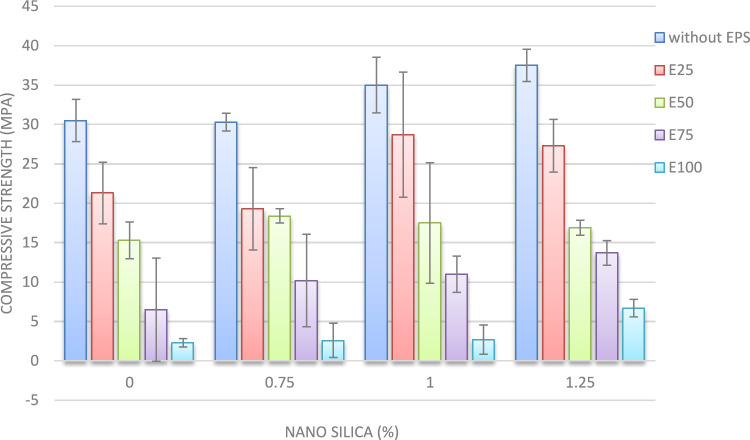
7-day compressive strength of mixes with different replacement of EPS with different percentage of NS.

**Fig. 16:**
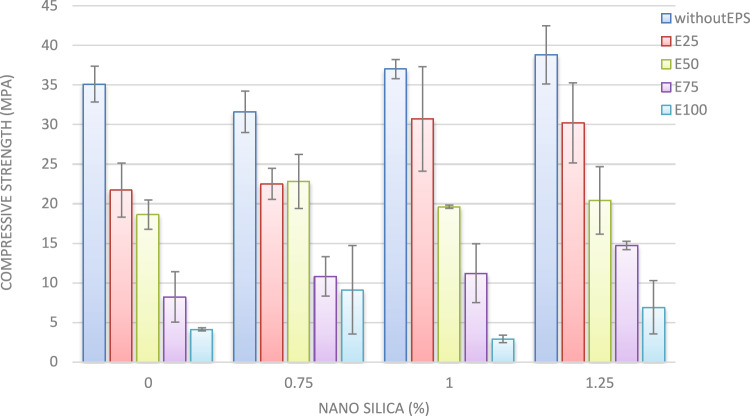
28-day compressive strength of mixes with different replacement of EPS with different percentage of NS.

The compressive strength results at 7 and 28 days are illustrated in Figs. 15 and 16. The incorporation of nano-silica into the concrete mixture notably enhances compressive strength. At 7 days, the addition of 1% and 1.25% nano-silica yielded increases in compressive strength of approximately 15% and 36.5%, respectively, compared to the control mix devoid of EPS and nano-silica. Conversely, the inclusion of 0.75% nano-silica resulted in a decrease in compressive strength of up to 10%.

At 28 days, the compressive strength of concrete increased by roughly 5% and 10% for mixes containing 1% and 1.25% nano-silica, respectively, in comparison to the control mix. These results underscore the positive impact of nano-silica on the long-term compressive strength of concrete.

Figure 15 provides a detailed analysis of the 7-day compressive strengths for mixes with varying nano-silica contents. In the mix containing 25% EPS, the addition of 0.75% nano-silica led to a 9% reduction in compressive strength. However, the incorporation of 1% and 1.25% nano-silica resulted in enhancements of 35% and 28%, respectively, relative to the mix with only 25% EPS. At the 28-day mark, compressive strength improvements of approximately 3%, 41%, and 39% were observed for the inclusion of 0.75%, 1%, and 1.25% nano-silica, respectively, when compared to the 25% EPS mix without nano-silica.

A similar trend was observed in the mixes containing 50% EPS, as depicted in Figs. 15 and 16. The addition of 0.75%, 1%, and 1.25% nano-silica enhanced the 7-day compressive strength by 20%, 14%, and 10%, respectively, compared to the mix with 50% EPS without nano-silica. At 28 days, the compressive strength improvements were approximately 22%, 5%, and 10% for the inclusion of 0.75%, 1%, and 1.25% nano-silica, respectively, compared to the mix with 50% EPS.

For mixes with 75% EPS, the presence of 0.75%, 1%, and 1.25% nano-silica significantly enhanced compressive strength at both 7 and 28 days. At 7 days, the improvements in compressive strength were 57%, 69%, and 111% for the inclusion of 0.75%, 1%, and 1.25% nano-silica, respectively, compared to the mix with 75% EPS without nano-silica. At 28 days, the corresponding improvements were approximately 32%, 36%, and 79%.

In the case of the mix with 100% EPS, the addition of 0.75%, 1%, and 1.25% nano-silica resulted in significant enhancements in compressive strength at 7 days, with improvements of 13%, 17%, and 191%, respectively, compared to the mix with 100% EPS without nano-silica. At 28 days, the enhancements in compressive strength were approximately 121% and 98% for the inclusion of 0.75% and 1.25% nano-silica, respectively. Notably, the incorporation of 1% nano-silica led to a 29% reduction in 28-day compressive strength compared to the mix with 100% EPS without nano-silica.

These findings highlight the beneficial effects of nano-silica on the compressive strength of lightweight concrete, with the extent of improvement being dependent on both the nano-silica content and the percentage of EPS replacement. The results offer valuable insights for optimizing mixture proportions and understanding the mechanical behavior of EPS lightweight concrete^30,31^.

#### Water permeability

**Fig. 17:**
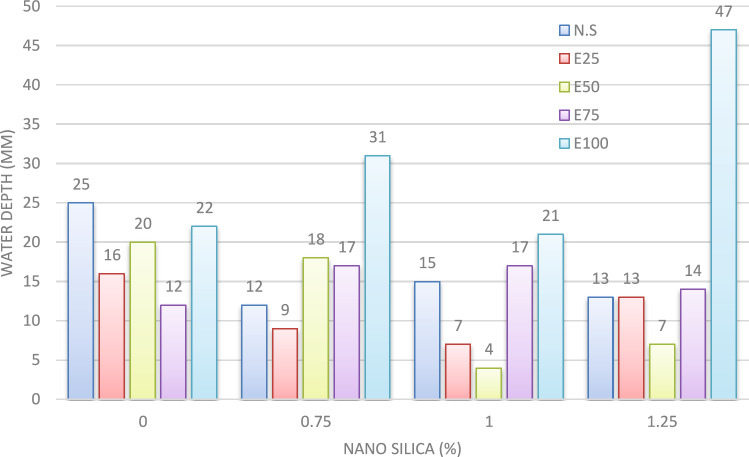
Water permeability for mixes with NS without EPS.

The water permeability of EPS concrete (EPS-C) is influenced by several factors, including cement content, concrete density, aggregate characteristics, and mineral admixtures. The experimental results illustrating the relationship between water permeability and the percentage of nano-silica are shown in Fig. 17. It is evident that the incorporation of 0.75%, 1%, and 1.25% nano-silica led to reductions in water permeability by approximately 52%, 40%, and 48%, respectively.

Additionally, the relationship between water permeability, nano-silica percentage, and EPS replacement is presented in Fig. 17. For the mix with a 25% EPS replacement, the use of nano-silica at 0.75%, 1%, and 1.25% resulted in decreases in water permeability of about 44%, 56%, and 19%, respectively. Similarly, in the mix with 50% EPS replacement, the addition of nano-silica at 0.75%, 1%, and 1.25% reduced water permeability by approximately 10%, 80%, and 65%, respectively.

However, in mixes with 75% EPS replacement, the effects of nano-silica on water permeability were varied. The inclusion of 0.75% nano-silica led to a decrease in water permeability of about 42%, while the use of 1% and 1.25% nano-silica resulted in increases of 42% and 17%, respectively.

Finally, in mixes with 100% EPS replacement, the presence of nano-silica resulted in increased water permeability. The addition of 0.75%, 1%, and 1.25% nano-silica increased water permeability by approximately 158%, 75%, and 291%, respectively, compared to the mix with only EPS.

These findings underscore the complex interplay between nano-silica content, EPS replacement, and water permeability in EPS-C. The inclusion of nano-silica can exert varying effects on water permeability, contingent upon the EPS content and nano-silica percentage employed. It is imperative to consider these factors when evaluating the water permeability characteristics of EPS-C for practical applications.

## Conclusions

The present study systematically investigated the effects of incorporating nano-silica and expanded polystyrene (EPS) on the thermal properties of concrete, as well as their influence on water absorption characteristics. The key findings are summarized as follows:

- *Thermal conductivity* The addition of nano-silica to concrete mixes exhibited a significant reduction in thermal conductivity. As the nano-silica content increased from 0.75% to 1.25%, the thermal conductivity coefficient decreased by up to 15%. This reduction can be attributed to several factors:

*Filler effect*: Nano-silica particles fill the voids between larger aggregates, creating a denser microstructure that reduces pathways for heat conduction.

*Pozzolanic reactivity* Nano-silica enhances the pozzolanic reactions within the concrete matrix, leading to the formation of additional calcium silicate hydrates (C–S–H) that improve the material’s overall thermal insulation properties.

*Air voids* The incorporation of nano-silica can also lead to increased air void content when being agglomerated especially at high dosages at certain locations within the matrix, which are effective thermal insulators.

- *Thermal diffusivity and volumetric heat capacity* Thermal diffusivity values decreased by up to 14% with increasing nano-silica content, while the volumetric specific heat capacity increased by up to 9% for mixes with 0.75–1% nano-silica. The underlying reasons include:

*Improved microstructure* The presence of nano-silica contributes to a more homogeneous and refined microstructure, which enhances the material’s ability to store and slowly transfer heat.

*Increased density* The additional C–S–H formation increases the density of the concrete, resulting in higher volumetric heat capacity, which allows the material to absorb and retain more heat without significant temperature changes.

- *Effect of EPS addition* The incorporation of EPS beads significantly enhanced the thermal insulation properties of concrete. As EPS content increased from 25 to 100%, thermal conductivity decreased by up to 80.5%. The observed effects can be explained as follows:

*Low density* EPS is a lightweight material that contains numerous air pockets, which effectively reduces thermal conductivity by providing paths of low thermal resistance.

*Structural integrity* However, at higher EPS replacements (E50 and E100), the volumetric heat capacity decreased, indicating a potential compromise in structural integrity and bonding within the concrete matrix. This might lead to increased voids and reduced inter-particle contact, impacting the overall performance.

- *Water absorption characteristics* The influence of EPS and nano-silica on water absorption was also observed. The decreasing trend in water absorption for E25 and E75 can be explained by:

*Increased density and reduced porosity* These mixes maintain sufficient density and packing, leading to fewer interconnected voids that allow water to permeate.

*Bonding characteristics* The combination of EPS and nano-silica may enhance bonding at these levels, further reducing water absorption.

Conversely, the increased water absorption in E50 and E100 might be due to:

*Higher void ratio* At these higher EPS contents, the concrete may develop a higher void ratio, making it more susceptible to water penetration.

*Altered microstructure* The excess EPS may disrupt the cement paste matrix, leading to a less cohesive structure that is more permeable.

Similarly, with varying nano-silica contents, a comparable trend was observed. Lower amounts of nano-silica enhance the densification of the matrix, while excessive amounts can lead to increased voids due to agglomeration or insufficient bonding with the cement matrix.

- *Synergistic effects of nano-silica and eps *The combined use of nano-silica (0.75–1.25%) and EPS (25–100%) resulted in a 39–86% reduction in thermal conductivity and significant reductions in thermal effusivity (28–71%) and diffusivity (28–79%). The synergistic effects can be attributed to:

*Complementary properties* Nano-silica enhances the structural integrity and thermal storage capacity, while EPS provides excellent insulation, leading to an overall improvement in thermal performance.

*Microstructural synergy* The combination creates a unique microstructure that optimizes thermal insulation while maintaining mechanical strength.

- *Mechanical and durability properties* The incorporation of nano-silica was found to enhance compressive strength and reduce water permeability. The optimal nano-silica content was identified as 1–1.25%, achieving compressive strength gains of up to 36.5% and a 40–52% reduction in water permeability due to:

*Enhanced C–S–H formation* The pozzolanic activity of nano-silica contributes to additional C–S–H formation, which fills voids and strengthens the concrete matrix.

*Improved bonding* This results in a denser and less permeable concrete structure, leading to improved durability.

In summary, the findings of this study provide valuable insights into the thermal, mechanical, and durability properties of concrete incorporating nano-silica and EPS. The synergistic effects of these additives, along with a detailed examination of their influence on water absorption, underscore the potential of this composite material for energy-efficient building applications, where enhanced thermal insulation, mechanical performance, and durability are critical requirements.

### Scalability and future work

While this composite shows transformative potential, industrial-scale production requires addressing:

- *Nano-silica Cost* Partnerships with suppliers for bulk procurement and in-situ synthesis methods are being explored.
- *EPS Integration* Pilot trials with recycled EPS waste (e.g., construction debris) have reduced raw material costs by 30%.
- *Long-Term Durability* Accelerated aging tests (100 + thermal cycles) are underway, with preliminary results indicating < 5% degradation in insulation properties.

## Data Availability

All data generated or analyzed during this study are included in this published article.
